# Self-Reported Walking Difficulty Associated with Stiff-Knee Gait in Japanese Patients with Knee Osteoarthritis: A Preliminary Cross-Sectional Study

**DOI:** 10.3390/healthcare9101308

**Published:** 2021-09-30

**Authors:** Haruki Toda, Tsubasa Maruyama, Koji Fujita, Yuki Yamauchi, Mitsunori Tada

**Affiliations:** 1 Digital Human Research Team, Artificial Intelligence Research Center, National Institute of Advanced Industrial Science and Technology, Tokyo 135-0064, Japan; tbs-maruyama@aist.go.jp (T.M.); m.tada@aist.go.jp (M.T.); 2 Department of Functional Joint Anatomy, Graduate School of Medical and Dental Sciences, Tokyo Medical and Dental University, Tokyo 113-8519, Japan; fujiorth@tmd.ac.jp; 3 Department of Orthopaedic Surgery, Doujin Hospital, Urasoe 901-2133, Japan; yamauchi.orth@tmd.ac.jp

**Keywords:** gait, inertial measurement unit, knee flexion excursion, knee osteoarthritis, motion capture, swing, walking difficulty

## Abstract

Background: Individuals with knee osteoarthritis are restricted in their daily activity because of walking difficulty. The purpose of this investigation was to examine the association between self-reported walking difficulty and knee flexion excursion during gait in Japanese patients with knee osteoarthritis. Methods: Twenty-eight patients with knee osteoarthritis participated in this study. Knee flexion excursions in loading response and swing during gait were measured through an inertial measurement unit-based motion capture system. The walking difficulty was assessed by a subitem in the Japanese Knee Osteoarthritis Measure. Pain intensity was assessed by a visual analog scale. Characteristics and gait variables were compared between groups that were determined a priori using the results of the walking difficulty assessment. The relationship between knee flexion excursion during gait and walking difficulty were analyzed using logistic regression. Results: The participants with walking difficulty had significantly small knee flexion excursion in both loading response and swing with large pain. After controlling the effect of pain, only knee flexion excursion in the swing was significantly related to the walking difficulty. Conclusions: This study suggested that the knee flexion excursion in swing during gait is helpful for understanding the walking difficulty experienced in Japanese patients with knee osteoarthritis.

## 1. Introduction

Knee osteoarthritis (OA) is a major musculoskeletal problem among the elderly. In Japan, the prevalence of radiographic knee OA has been reported to be 48.2% in males and 71.9% in females aged 70–79 years [1]. Knee OA limits the activities of daily living [2]. Furthermore, knee OA is a potentially modifiable risk factor for serious cardiovascular diseases and death [3]. To reduce the risk of developing functional limitations, people with knee OA are recommended to walk over 6000 steps/day [4]. In addition, walking for at least 30 min is considered a moderate aerobic exercise that can help to prevent the progression of knee OA [5]. Although walking is a simple motion that can be performed in daily living, many individuals with knee OA avoid walking during their daily activities due to knee pain and stiffness [6,7]. Therefore, to promote walking, the perceived difficulty among patients with knee OA should be minimized.

Walking difficulty in Japanese patients with knee OA should be assessed according to their lifestyle and sense of values. Previous studies reported by Na et al. [8,9] examined walking difficulty in patients with knee OA using a subitem of the Knee Outcome Survey (KOS). However, the results of this evaluation may be influenced by differences in cross-cultural backgrounds if adapted to knee OA among Japanese people. Therefore, Akai et al., [10] developed the Japanese Knee Osteoarthritis Measure (JKOM) based on the International Classification of Functioning, Disability, and Health. JKOM subitems also have questions regarding walking difficulty on flat surfaces.

Individuals with knee OA exhibit a stiff-knee gait pattern characterized by small knee flexion excursion during the loading response and swing phase [11,12]. Previous studies have reported the association of knee kinematics and kinetics during the early stance of walking with the self-reported walking difficulty evaluated by KOS among individuals with knee OA [8,9]. However, to our knowledge, no study has examined the relationship between self-reported walking difficulty evaluated by JKOM and knee flexion excursion in both loading response and swing among Japanese patients with knee OA.

The stiff-knee gait pattern is caused by abnormal muscle activation around the knee [13] and increases mechanical stress in the knee [14]. Knee flexion excursion in the loading response contributes to attenuating the impact load on the articular cartilage by weight acceptance. Additionally, in the swing phase, the knee flexion motion to ensure toe clearance is required to avoid the risk of falling, especially on an uneven surface in the outdoor environment. Moreover, inadequate knee flexion in the swing induces energy-inefficient compensatory movements [15]. Stiff-knee gait patterns may hence contribute to the self-reported walking difficulty among individuals with knee OA.

This study aimed to examine the relationship between self-reported walking difficulty and knee flexion excursion in loading response and swing among Japanese patients with knee OA. We hypothesize that participants with small knee flexion excursions during gait show a higher possibility of experiencing walking difficulties in their daily living than those with large knee flexion excursions.

## 2. Materials and Methods

### 2.1. Participants

Individuals with knee OA participated in this study. All participants were diagnosed with tibiofemoral knee OA through an evaluation using weight-bearing anteroposterior radiographs with the Kellgren–Lawrence (K-L) classification of grade 1 or higher in addition to clinical symptoms. The participants could walk independently without any assistive devices. If the participant had knee OA on both lower limbs, we selected the more symptomatic side. The exclusion criteria included neurologic disorders, trauma, and a history of knee surgery.

### 2.2. Clinical Evaluation

Knee OA severity was assessed using the K-L grade [16]. K-L grade and femorotibial angle (FTA) were evaluated using weight-bearing anteroposterior radiographs.

The participants completed a self-administered questionnaire using the visual analog scale (VAS) as well as the JKOM [10]. VAS was used to evaluate the degree of pain in the knee during walking on a scale of 0–100 mm. Walking difficulty was assessed using the JKOM subitem regarding walking on a flat surface: “How difficult can you walk on a flat surface without taking a rest? (0 = over 30 min, 1 = about 15 min, 2 = around your house, 3 = only into your house, 4 = almost not)”. Participants who scored 0 were assigned into the non-difficulty (non-Diff) group, whereas participants who scored ≥ 1 were assigned into the difficulty (Diff) group. 

### 2.3. Range of Motion

A plastic goniometer (OG wellness Co., Ltd, Okayama, Japan) was used to measure the passive knee extension range of motion (RoM) in 5° increments. The participants were examined in the supine position on an examination table. The goniometer was aligned over the sagittal axis of the thigh and shank.

### 2.4. Muscle Strength

A hand-held dynamometer (HHD) (μTas F-1; Anima Corp., Tokyo, Japan) was used to measure maximum isometric knee extension and flexion muscle strength. The participants were examined in a sitting position on an examination table with the hip and knee joints at 90° flexion, the lower leg perpendicular to the floor and the feet not touching the floor, according to a previous study [17]. The HHD sensor was attached to in front of and behind the distal end of the shank during extension and flexion, respectively. Measurements were taken for 5 s each after the practice trials. The measured forces were normalized by shank length and body weight (Nm/kg).

### 2.5. Gait Measurement

Whole-body motion during walking was measured using an inertial measurement unit (IMU)-based motion capture system. This system was developed by Maruyama et al. [17] and validated in our previous studies [18,19]. Thirteen IMUs (MTw; Xsens Technologies Inc., Enschede, The Netherlands) were attached to each body segment of the participants in accordance with our previous study [20]. The participants were allowed to use their shoes during walking to ensure high fidelity in measuring the knee’s natural function [10]. A reference pose was taken prior to the walking trials for calibration of the IMU orientation to the corresponding body segment. Afterwards, the participants walked approximately 12 m in a straight walkway at the hospital with a self-selected comfortable walking speed.

### 2.6. Data Analysis

A posture-reconstruction plugin running on DhaibaWorks, a self-developed motion analysis software described previously [21], was used for the knee joint angle calculation at 60 Hz. This plugin reconstructed the lower limb motion by combining the orientation of IMUs attached to body segments and the individual body model through a link structure. The dimensions of the model have been estimated statistically from the participants’ body height and weight based on the Japanese body dimensions database [20]. In addition, we reflected participants’ FTA and passive knee extension RoM in the body models.

**Figure 1:**
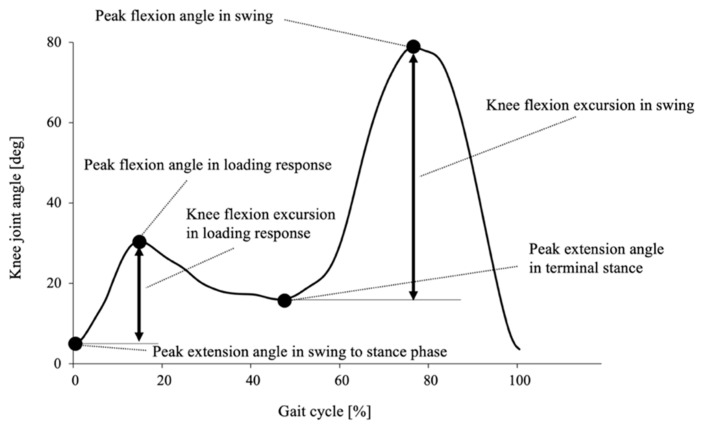
Typical knee flexion angle of a subject in one gait cycle. The knee flexion excursions were calculated from the amplitude of displacement from the peak extension angle to the peak flexion angle during the loading response and swing phase.

A Butterworth low-pass filter at 6 Hz was applied to the kinematic data. Data for five gait cycles during steady-state walking were extracted. The knee flexion excursions in the loading response and swing were calculated from the displacements between the maximum extension angle around the initial contact and the maximum flexion angle in the loading response, and the maximum extension angle in the terminal stance and the maximum flexion angle in the swing phase, respectively (Figure 1). The stride length was calculated as the distance between the heel points projected on the sagittal plane. The walking speed was calculated from the stride length and the duration of one gait cycle time.

### 2.7. Statistical Analysis

We used the Mann–Whitney U-test to examine continuous variables and the chi-square test to examine categorical variables between the non-Diff and Diff groups.

Logistic regression analysis was conducted with walking difficulty (non-difficulty or difficulty) as the dependent variable. We created two logistic regression models to determine whether knee flexion excursion in loading response (Model 1) and swing (Model 2) affects walking difficulty. In addition, the variables that differed between the non-Diff and Diff groups were entered into each model as an adjustment variable using forced entry.

The significance level was set at 0.05. All data were analyzed using the SPSS statistical software version 25.0 (IBM, Armonk, NY, USA). 

## 3. Results

Twenty-eight patients with knee OA participated in this study. Nineteen participants were classified into the non-Diff group, whereas nine participants were classified into the Diff group (walking on a flat surface; JKOM scores were as follows: score of 1 = five participants, score of 2 = one participants, score of 3 = three participants). Table 1 shows the characteristics of the participants in each group.

**Figure 2:**
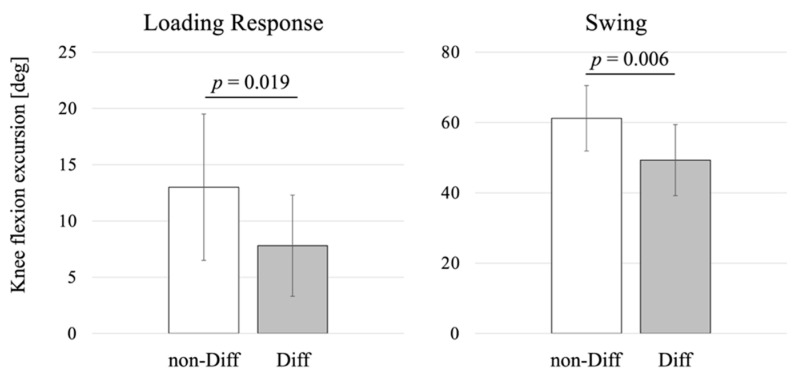
Comparison of knee flexion excursions in loading response and swing between the groups with walking difficulty (Diff) and without walking difficulty (non-Diff).

There were no significant differences in age, sex, height, weight, body mass index, K-L grade, muscle strength, and walking speed between the non-Diff and Diff groups. In contrast, the Diff group had significantly smaller knee flexion excursions in both loading response and swing, with larger VAS score during walking than the non-Diff group (Table 1 and Figure 2).

Table 2 shows the results of the logistic regression analysis. Because the VAS scores were significantly different between the groups, this variable was entered into the logistic regression models, with the knee flexion excursions in loading response and swing. As a result, the knee flexion excursion in the swing was observed to be significantly related to walking difficulty. In contrast, the knee flexion excursion in the loading response was not statistically significant after controlling for the effect of the VAS walking.

## 4. Discussion

**Figure 3:**
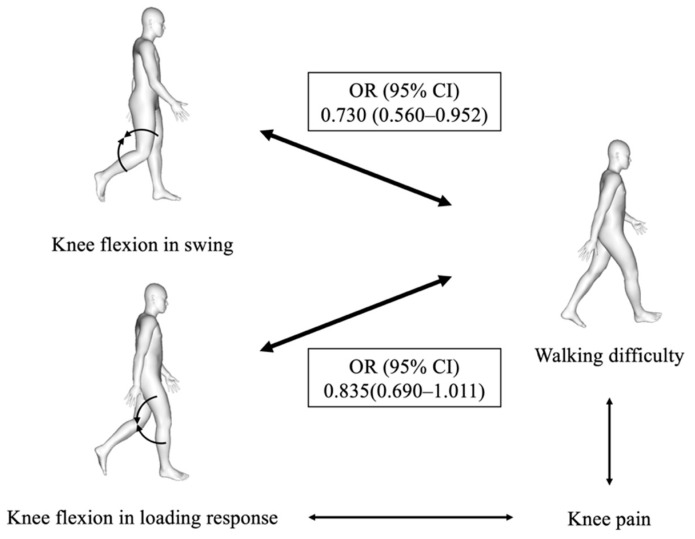
Graphical abstract of this study. Self-reported walking difficulty in Japanese patients with knee osteoarthritis was associated with the knee flexion excursion in the swing. The knee flexion excursion in the loading response is not related to the walking difficulty after controlling for the factor of knee pain. Odds ratios (OR) with their 95% confidence intervals (CI) are provided.

This study examined whether the stiff-knee gait pattern of individuals with knee OA was related to self-reported walking difficulty in daily living, and we found that the knee flexion excursion in the swing was associated with difficulty in walking on a flat surface (Figure 3), which partially supports our hypothesis.

Knee flexion motion in the swing was significantly related to the difficulty of walking on a flat surface, despite controlling for the factor of knee pain. The present study showed that the individuals with small knee flexion excursion in the swing were likely to experience walking difficulty in daily living, which could imply that this motion is helpful for understanding the restrictions in walking duration and environment regardless of the knee pain. Generally, a small knee flexion motion in the swing is caused by the overactivity of the quadriceps [22]. This motion controls the toe clearance [23]. Individuals with knee OA who had a small knee flexion excursion in the swing have a large stumbling risk because of the difficulty in adapting to changes in the environment [24]. Avoiding stumbling, these individuals adopted to the compensatory trunk and lower limb movements [25]. In addition, the overactivity and compensatory movements caused by stiff-knee gait patterns lead to higher energy costs [26]. A previous study reported that a small range of knee flexion–extension in a gait cycle was correlated with a short six-minute walk distance in individuals with knee OA [27]. Decreasing the knee flexion in the swing might also reduce walking activity in terms of energy costs and the risk of stumbling. This suggests that interventions for the inhibition of the activity of the quadriceps in the terminal stance and swing assistance using a wearable pneumatic artificial muscle [28] might effectively increase the walking activity by minimizing the walking difficulty in individuals with knee OA, and further research is warranted.

The Diff group had a small knee flexion excursion during the loading response. In contrast, a previous study by Na et al. [8] reported that the knee flexion excursion in the loading response did not differ between patients with and without walking difficulty, which is inconsistent with our results. There may be a few reasons for this difference. The previous study evaluated the walking difficulty using a KOS, which asked participants to feel the difficulty while walking. In contrast, our study employed the JKOM, which asked about the participants’ walking activity in daily life, such as the duration and environment. The contents of the respective questions may have affected the participant grouping. In addition, although Na et al. [8] controlled the subjects’ walking speed, our participants walked at a comfortable speed. The different experimental designs may have also affected the results. Nevertheless, our study results suggest that the relationship between the walking difficulty and the knee flexion excursion in the loading response was small, based on logistic regression analysis.

The participants in the Diff group had significantly higher VAS score than those in the non-Diff group. Pain and its related fear are characterized by escape and avoidance behaviors, and avoidance of daily activities results in functional disability [29]. The JKOM assessed the walking difficulty by asking about the walking duration and environment in daily living. Since participants in the Diff group experienced considerable pain, their walking duration and environment could be restricted by the pain-related avoidance of daily activities. Moreover, knee pain induces small knee excursions to reduce the external force generated in the knee joint [11]. Knee pain affected the relationship between the walking difficulty and small knee excursions in the loading response as a confounding variable. Although the participants with a small knee flexion excursion in the loading response felt that walking difficulty, the effect on walking difficulty was small when controlling for the factor of knee pain.

This study had some limitations. First, walking difficulty was evaluated using a self-administrated questionnaire. Patient-reported outcome measures can be easily evaluated in a clinical setting. On the other hand, walking area and time and the frequency of use of a cane can be quantitatively measured using a wearable sensor, that is, an IMU and a pedometer. Further studies are needed to examine the relationship between walking activity in daily living as measured by wearable sensors and joint kinematics during walking. Second, this study employed a cross-sectional design. Therefore, we could not identify a causal relationship between walking difficulty and knee flexion excursion based on the results. Third, this study is a preliminary study with a small sample size. To generalize the results of this study, a homogenized group with a larger sample size is necessary. Finally, in this study, no evaluations were performed using the Western Ontario and McMaster Universities Osteoarthritis Index and Knee Outcome Survey, which are international knee OA scales. Thus, we cannot compare the results of this study to studies that used the international scales.

## 5. Conclusions

This study revealed that individuals with knee OA exhibit the stiff-knee gait pattern, wherein the small knee flexion excursion in the swing was associated with the self-reported walking difficulty in daily living. The results suggest that to increase their walking activity, interventions to increase knee flexion excursion in the swing should be taken into consideration.

## Figures and Tables

**Table 1:** Characteristics and outcome measures of the groups with walking difficulty (Diff) and without walking difficulty (non-Diff).

Characteristics	Non-Diff Group (*n* = 19)	Diff Group (*n* = 9)	*p*-Value
Age (years)	72.8 ± 10.1	72.8 ± 11.5	0.847
Sex (*n*)			0.483
Male	4 (21.1%)	3 (33.3%)	
Female	15 (78.9%)	6 (66.7%)	
Height (m)	1.53 ± 0.09	1.54 ± 0.11	0.847
Weight (kg)	58.7 ± 9.7	58.8 ± 8.6	0.885
BMI (kg/m^2^)	24.8 ± 3.1	24.7 ± 3.0	0.962
K-L grade (*n*)			0.259
Grade I	4 (21.1%)	1 (11.1%)	
Grade II	7 (36.8%)	1 (11.1%)	
Grade III	4 (21.1%)	5 (55.6%)	
Grade Ⅳ	4 (21.1%)	2 (22.2%)	
Muscle strength (Nm/kg)			
Flexion	0.66 ± 0.18	0.54 ± 0.11	0.142
Extension	1.09 ± 0.28	0.90 ± 0.24	0.085
VAS (mm)	37.0 ± 31.8	58.7 ± 27.6	0.010 *
Walking speed (m/s)	1.18 ± 0.32	0.98 ± 0.24	0.156

Value: Mean ± standard deviation. * *p* < 0.05. BMI, body mass index; K-L, Kellgren–Lawrence grade; VAS, visual analog scale.

**Table 2:** Results of the logistic regression analysis between the groups with walking difficulty (Diff) and without walking difficulty (non-Diff).

Variables		OR ^1^	95% CI ^2^		*p*-Value
			Lower	Upper	
Model 1	Knee flexion excursion in loading response	0.835	0.690	1.011	0.064
	VAS ^3^	1.049	1.005	1.085	0.027 *
Model 2	Knee flexion excursion in swing	0.730	0.560	0.952	0.020 *
	VAS ^3^	1.086	1.010	1.154	0.025 *

* *p* < 0.05. ^1^ OR, odds ratio; ^2^ CI, confidence interval; ^3^ VAS, visual analog scale.

## Data Availability

Data sharing not applicable.
